# Effectiveness of Differentiating Mold Levels in Soybeans with Electronic Nose Detection Technology

**DOI:** 10.3390/foods13244064

**Published:** 2024-12-17

**Authors:** Xuejian Song, Lili Qian, Dongjie Zhang, Xinhui Wang, Lixue Fu, Mingming Chen

**Affiliations:** 1 College of Food Science, Heilongjiang Bayi Agricultural University, Daqing 163319, China; byndsxj@126.com (X.S.); w604466213@163.com (X.W.); wxh89757@gmail.com (L.F.); chenmingming515@163.com (M.C.); 2 Key Laboratory of Agro-Products Processing and Quality Safety of Heilongjiang Province, Daqing 163319, China; 3 National Coarse Cereals Engineering Research Center, Daqing 163319, China

**Keywords:** soybean, electronic nose, mildew, principal component analysis, linear discriminant analysis

## Abstract

This study employed electronic nose technology to assess the mold levels in soybeans, conducting analyses on artificially inoculated soybeans with five strains of fungi and distinguishing them from naturally moldy soybeans. Principal component analysis (PCA) and linear discriminant analysis (LDA) were used to evaluate inoculated and naturally moldy samples. The results revealed that the most influential sensor was W2W, which is sensitive to organic sulfur compounds, followed by W1W (primarily responsive to inorganic sulfur compounds), W5S (sensitive to small molecular nitrogen oxides), W1S (responsive to short-chain alkanes such as methane), and W2S (sensitive to alcohols, ethers, aldehydes, and ketones). These findings highlight that variations in volatile substances among the moldy soybean samples were predominantly attributed to organic sulfur compounds, with significant distinctions noted in inorganic sulfur, nitrogen compounds, short-chain alkanes, and alcohols/ethers/aldehydes/ketones. The results of the PCA and LDA analyses indicated that while both methods demonstrated moderate effectiveness in distinguishing between different dominant fungal inoculations and naturally moldy soybeans, they were more successful in differentiating various levels of moldiness, achieving a discriminative accuracy rate of 82.72% in LDA. Overall, the findings suggest that electronic nose detection technology can effectively identify mold levels in soybeans.

## 1. Introduction

Soybeans (*Glycine max* (L.) Merr.), an annual herbaceous plant in the legume family, serve as a vital source of nutrition and are a primary provider of plant-based protein and oils [1]. They contain approximately 40% protein with an amino acid profile beneficial to human health, 20% cholesterol-free oil comprising a high proportion of unsaturated fatty acids, and 33% carbohydrates, along with various essential minerals [2]. Additionally, soybeans are rich in bioactive compounds, including oligosaccharides, saponins, isoflavones, phytosterols, trypsin inhibitors, and bioactive peptides [3,4,5,6,7,8]. China stands out as a major producer and importer of soybeans. To ensure a stable market supply, large quantities of soybeans are transported across regions annually. However, the complex transportation environment often leads to spoilage. Previous studies have identified several fungi, including *Aspergillus*, *Fusarium*, *Penicillium*, *Cladosporium*, *Mucor*, and *Rhizopus*, as the primary agents of soybean spoilage [9]. Spoiled soybeans exhibit characteristics such as dark, grayish discoloration, a musty odor, reduced dispersibility, decreased nutrient content, altered composition, and degraded processing and food quality. Therefore, the early detection of soybean spoilage and the timely implementation of control measures are essential to mitigating economic losses.

The growth metabolism and extensive proliferation of mold in grains can lead to surface damage and discoloration, accelerate the loss of dry matter, and degrade starch, protein, and lipids while altering digestibility. Additionally, mold contamination can produce off-flavors, significantly reducing grain quality [10]. Current methods for detecting fungal contamination in grains are categorized into traditional detection methods (such as microbiology [11], high-performance liquid chromatography (HPLC) [12], thin-layer chromatography (TLC) [13], enzyme-linked immunosorbent assays (ELISA) [14], adenosine triphosphate (ATP) bioluminescence [15], and polymerase chain reaction (PCR) [16]) and non-destructive detection methods (including imaging technology, spectroscopic techniques, electronic nose (E-nose), and gas chromatography–ion mobility spectrometry (GC-IMS)). Traditional detection methods yield precise and reliable results; however, they often require cumbersome sample preparation, complex extraction procedures, and lengthy quantitative analyses. These factors can result in sample loss and hinder the rapid analysis of large-scale samples. In contrast, Non-destructive detection methods are increasingly used in grain mold detection due to their simplicity, efficiency, and high accuracy. Shen et al. [17] combined near-infrared (NIR) spectroscopy with computer vision for the online detection of moldy corn kernels, achieving a classification accuracy of 100% for corn samples contaminated with different molds. Siripatrawan et al. [18] developed hyperspectral imaging (HSI) technology for detecting mold contamination in stored rice, utilizing self-organizing maps (SOM) for the visual classification of data from samples with varying degrees of spoilage, confirming the effectiveness of HSI for the early detection of mold contamination in stored rice. Gu et al. [19] employed GC-IMS to detect moldy wheat samples and conducted validation experiments to simulate aflatoxin infection rates in field samples, incorporating both non-targeted spectral fingerprints and targeted specific markers as chemometric strategies to assess the performance of classification and prediction models. Wang et al. [20] utilized GC-IMS combined with fluorescence spectroscopy for the rapid detection of potential aflatoxins in peanut kernels. This study introduced data-level and feature-level fusion strategies, using the first ten principal components combined with orthogonal partial least squares discriminant analysis (OPLS-DA) for feature-level data fusion, providing a more accurate characterization of healthy and moldy peanuts (96.7%). Simultaneously, regression models predicting total aflatoxin levels in peanuts were established based on independent and fused signals using partial least squares regression (PLSR). The results indicate that the fusion of GC-IMS and fluorescence spectroscopy can effectively monitor aflatoxins in peanuts, offering the potential for the early warning of aflatoxin contamination.

The electronic nose (E-nose) is a detection system that simulates mammalian olfaction by generating unique composite responses to odors [21]. It comprises three main components: a sample processing system, an array of electrochemical gas sensors, and a pattern recognition system, The E-nose is typically employed for detecting both simple and complex volatile organic compounds [22]. The sensor array can consist of multiple sensing elements, a single device, or a combination of both [23]. The thin film of the sensor element undergoes chemical reactions with sampled molecules, and the resulting electronic conversions are detected by the sensor as electrical signals [24]. Spoilage can cause surface damage and discoloration of the grain, accelerate dry matter loss, and produce off-flavors, significantly degrading its quality [25]. E-nose technology, owing to its high sensitivity to odors, is widely used in food quality and safety monitoring [26]. Compared to traditional methods, E-nose technology offers advantages such as cost-effectiveness, efficiency, and strong adaptability. Roberto et al. [27] utilized the E-nose to detect volatile compounds in mold-infected wheat seeds, differentiating between spoiled samples (contaminated with *Aspergillus flavus* and *Fusarium verticillioides*) and healthy wheat samples, confirming the feasibility of the E-nose for detecting fungal infections in wheat. Mota et al. [28] demonstrated that the E-nose could provide satisfactory indicators of contamination rates. To enhance the accuracy of discrimination results, researchers have begun integrating chemometric methods for analytical recognition. Ying et al. [29] investigated an early spoilage prediction method for grains based on the E-nose and probabilistic neural networks (PNN), measuring and recording the E-nose responses of rice, red beans, and oats at varying spoilage levels while employing principal component analysis (PCA), backpropagation (BP) networks, and PNN for the discriminative analysis of the detection data. The results showed that PCA and BP networks struggled to differentiate among the three-grain samples at different spoilage levels, exhibiting poor predictive accuracy, while PNN demonstrated strong discrimination capability, achieving an accuracy rate of 93.75%, indicating that the E-nose and PNN approach can effectively predict early grain spoilage. However, the performance of the E-nose still faces limitations, as factors such as drift, environmental influences, sensor redundancy, and the signal-to-noise ratio can affect detection results [30]. Therefore, to provide a more comprehensive description of volatile compounds, researchers have begun combining the E-nose with other non-destructive techniques. Shen et al. [17] conducted the rapid detection of aflatoxin contamination levels in peanuts using near-infrared (NIR) and E-nose technology, collecting feature information at different storage stages (0, 3, 6, and 9 d) and classifying peanut samples of varying contamination levels using linear discriminant analysis (LDA), achieving discrimination accuracies of 92.11% and 86.84% for NIR and E-nose, respectively. They also quantified the total fungal count in peanut samples using partial least squares regression (PLSR), obtaining good predictive results. Gu et al. [31] combined the E-nose with pattern recognition algorithms to effectively detect mold contamination levels in rice, preventing spoiled rice from entering the food chain and confirming the feasibility of integrating the E-nose with pattern recognition algorithms for detecting fungal contamination in grains [32].

This study employed electronic nose (E-nose) technology to detect volatile compounds in both artificially inoculated and naturally spoiled soybean samples. The analysis focused on sensor response values and contribution rates to explore the concentration changes of various volatile substances during soybean spoilage and to classify different spoilage levels. This study is crucial for the early warning and rapid detection of grain spoilage using E-nose technology.

## 2. Materials and Methods

### 2.1. Preparation of Mold Spore Suspension

Based on preliminary experimental results [33], five strains of fungi, *Aspergillus niger*, *Penicillium rubens*, *Rhizopus microsporus*, *Penicillium oxalicum*, and *Aspergillus versicolor*, were isolated from the surface of moldy soybeans. The fungal strains were activated in Bengal rose agar medium (Shanghai Bowei Biotechnology Co., Ltd., Shanghai, China) at 28 °C for 3 to 7 d to produce a substantial quantity of spores. Subsequently, the strains were eluted using 0.9% sterile saline, with an inoculating spatula used to gently scrape the surface of the culture to ensure effective elution. The elution was filtered through two layers of sterile medical gauze to remove mycelium and other impurities [34], and the spore suspension was counted using a hemocytometer (25 × 16), adjusting the concentration to 1 × 10^6^ to 1 × 10^7^ CFU/mL for subsequent use.

### 2.2. Inoculation and Incubation

The soybean used in this experiment is the dominant variety Haiong 48, which was harvested in Qiqihar, Heilongjiang Province, China. Haiong 48 soybeans feature plump grains with a shiny yellow seed coat, a yellow hilum, and an average weight of approximately 22.0 g per hundred grains. To minimize the influence of other fungi on the results, the soybean samples were sterilized by irradiation before the experiment to ensure sterility. Following the method of Zhang et al. with slight modifications [10], a dosage of 10 kGy γ-rays was applied for 10 h of irradiation, with samples agitated every 30 min to maximize sterility.

A spore suspension of the five fungal strains was evenly sprayed on 500 g of soybean samples at a 1:10 mass ratio, while another 500 g of non-irradiated dew-soaked soybean samples were placed in a climate chamber for cultivation (40 °C, RH = 90%). Random sampling was conducted at 0 (control group), 8, 16, and 24 d, with three samples of 10 g taken from each group, following GB 4789.15-2016 food safety national standards for the microbiological examination of foods to determine the total fungal colony count in the soybean samples [35]. The grain safety levels adhered to LS/T6132-2018 for the detection of stored grain fungi by the spore counting method [36], where non-spoiled soybeans had fungal counts <1.0 × 10^5^ CFU/g, mild spoilage was 1.0~9.9 × 10^5^ CFU/g, moderate spoilage was 1.0~9.9 × 10^6^ CFU/g, and severe spoilage was >1.0 × 10^7^ CFU/g. The soybean samples were then placed in 100 mL quartz beakers, sealed with double-layer sealing film, and allowed to stand at room temperature for 30 min to equilibrate before conducting E-nose measurements.

### 2.3. E-Nose Detection

The PEN3 electronic nose (AIRSENSE Technologies, Schwerin, Germany) is equipped with ten different built-in metal oxide gas sensors capable of detecting and identifying various common gases (Table 1), allowing for the capture of the characteristic aroma of soybeans [37]. It consists of three main components: a data acquisition, modulation, and transmission unit; a sensor array and chamber unit; and a power and gas supply system. The sampling needle is inserted into a sealed beaker containing equilibrated moldy soybean samples for measurement under the following conditions: sampling time of 1 s; self-cleaning time of 80 s; zeroing time of 5 s; sample preparation time of 5 s; injection flow rate of 400 mL/min; and analysis sampling time of 80 s.

### 2.4. Data Analysis

Data were analyzed for variance and correlation using IBM SPSS Statistics 26 software, while principal component analysis (PCA) and linear discriminant analysis (LDA) were conducted using the WinMuster software (v. 1.2) included with the PEN3 electronic nose.

## 3. Results and Analysis

### 3.1. Sensor Response Analysis of Mold Inoculation and Natural Mildew Soybean

**Figure 1:**
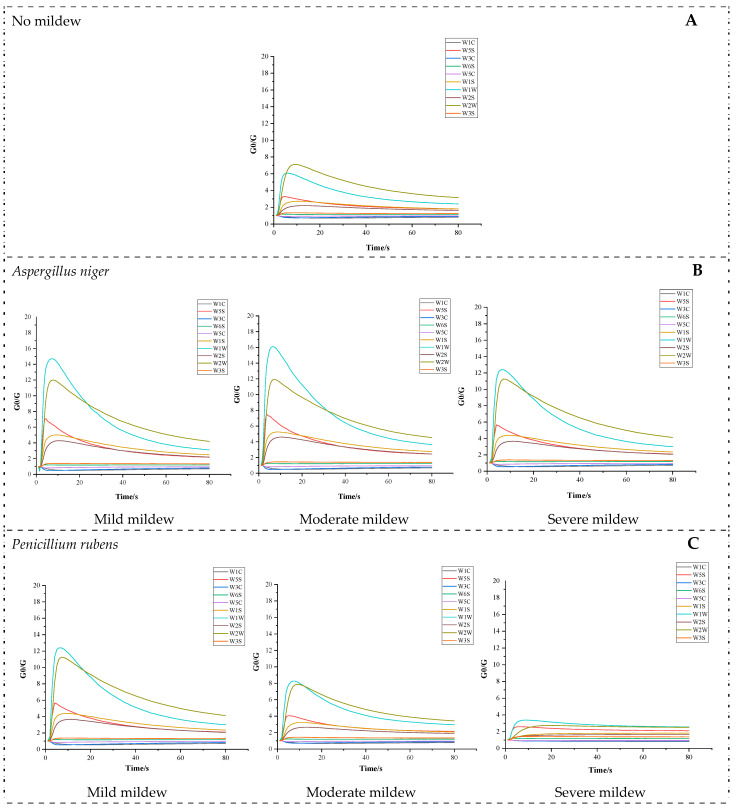

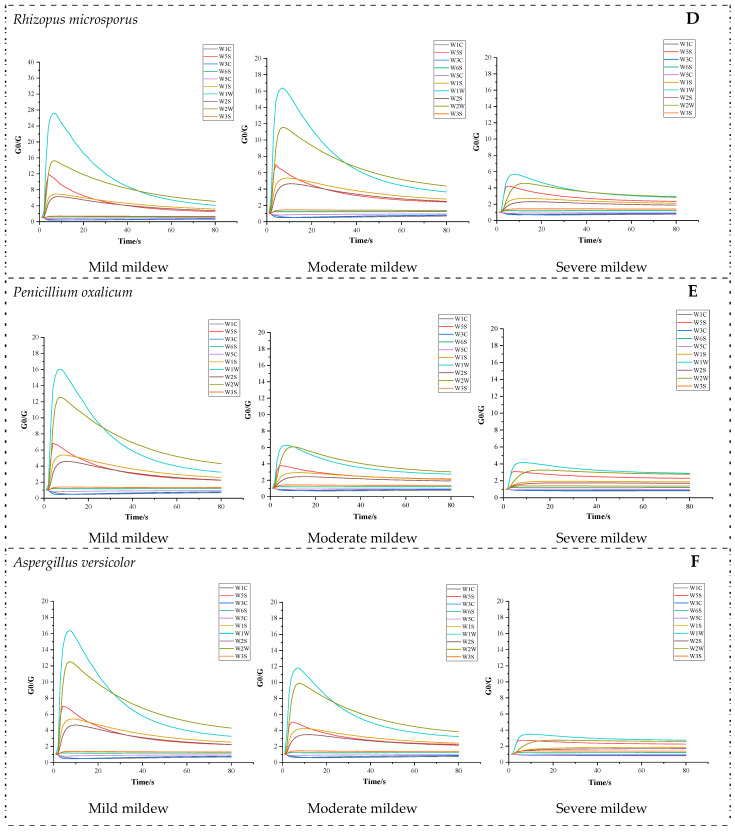

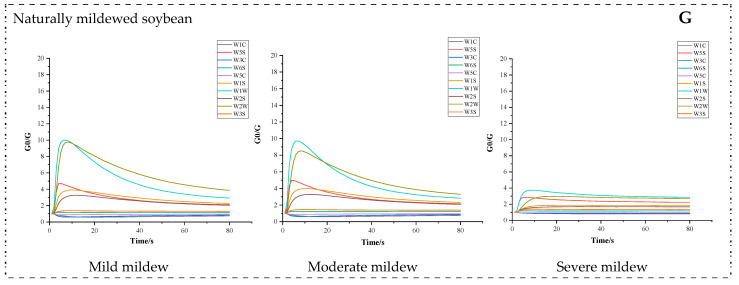
Analysis of the response value of sensors for each mildew degree. (**A**) No mildew; (**B**) *Aspergillus niger*; (**C**) *Penicillium rubens*; (**D**) *Rhizopus microsporus*; (**E**) *Penicillium oxalicum*; (**F**) *Aspergillus versicolor*; (**G**) Naturally mildewed soybean.

The response curve of the metal oxide semiconductor (MOS) sensor comprises three phases: the initial phase, the change phase, and the stabilization phase [38]. Figure 1 illustrates the analysis of sensor response values for five types of mold-inoculated and naturally moldy soybean samples, with the x-axis representing the detection time (0–90 s) and the y-axis representing the G_0_/G value (initialized to 1), where G and G_0_ are the resistance values of the sensor when detecting the sample gas and clean air, respectively [39]. Each curve depicts the response values of each sensor over time. According to the response mechanism of the MOS sensor, variations in the sensor response values are correlated with the concentration of volatile compounds [40]. By observing the response levels of the ten sensors, differences in the concentration of volatile substances in moldy soybean samples can be analyzed.

#### 3.1.1. *Aspergillus niger*

The analysis of sensor response values for moldy soybean samples inoculated with *Aspergillus niger* at various mold levels is presented in Figure 1A. Overall, the sensors exhibiting the highest response values are W1W (sensitive to inorganic sulfides) and W2W (sensitive to organic sulfides), followed by W5S (highly sensitive to nitrogen oxides), W1S (sensitive to short-chain alkanes such as methane), and W2S (sensitive to alcohols, ethers, aldehydes, and ketones). Notably, W1W and W2W demonstrate the most pronounced responses in the cases of mild and moderate mold. This indicates that the concentrations of organic sulfides, inorganic sulfides, and nitrogen compounds are relatively high in the moldy soybean samples inoculated with *Aspergillus niger*.

#### 3.1.2. *Penicillium rubens*

Figure 1B presents the analysis of sensor response values for moldy soybean samples inoculated with *Penicillium rubens* at various mold levels. It can be observed that the sensors with the highest overall response values are W1W (sensitive to inorganic sulfides) and W2W (sensitive to organic sulfides), followed by W5S (highly sensitive to nitrogen oxides). However, under severe mold conditions, the responses of all sensors are relatively weak, with deviation values less than 4. This indicates that while the concentrations of organic sulfides, inorganic sulfides, and nitrogen compounds are relatively high in the moldy soybean samples inoculated with *Penicillium rubens*, they are comparatively low under severe mold conditions.

#### 3.1.3. *Rhizopus microsporus*

As shown in Figure 1C, under mild and moderate mold conditions, the sensors with the highest response values are W1W (sensitive to inorganic sulfides) and W2W (sensitive to organic sulfides). These are followed by W5S (highly sensitive to nitrogen oxides), W1S (sensitive to short-chain alkanes such as methane), and W2S (sensitive to alcohols, ethers, aldehydes, and ketones). This indicates that the relative concentrations of organic sulfides, inorganic sulfides, and nitrogen compounds are higher in the moldy soybean samples inoculated with *Rhizopus microsporus* under mild and moderate mold conditions. However, under other conditions, the concentrations of these substances are relatively low.

#### 3.1.4. *Penicillium oxalicum*

Figure 1D presents the analysis of sensor response values for moldy soybean samples inoculated with *Penicillium oxalicum* at various mold levels. Under mild mold conditions, the sensors with the highest response values are W1W (sensitive to inorganic sulfides) and W2W (sensitive to organic sulfides). These are followed by W5S (highly sensitive to nitrogen oxides), W1S (sensitive to short-chain alkanes such as methane), and W2S (sensitive to alcohols, ethers, aldehydes, and ketones). However, under other conditions, the sensor response values are generally low. This indicates that under mild mold conditions, the concentrations of organic sulfides, inorganic sulfides, and nitrogen compounds are relatively high in the moldy soybean samples inoculated with *Penicillium oxalicum*. Conversely, under other conditions, the concentrations of these substances are relatively low.

#### 3.1.5. *Aspergillus versicolor*

Figure 1E presents the analysis of sensor response values for moldy soybean samples inoculated with *Aspergillus versicolor* at various mold levels. Under mild and moderate mold conditions, the sensors with the highest response values are W1W (sensitive to inorganic sulfides) and W2W (sensitive to organic sulfides). These are followed by W5S (highly sensitive to nitrogen oxides), W1S (sensitive to short-chain alkanes such as methane), and W2S (sensitive to alcohols, ethers, aldehydes, and ketones). This indicates that in the moldy soybean samples inoculated with *Aspergillus versicolor*, the relative concentrations of organic sulfides, inorganic sulfides, and nitrogen compounds are higher under mild and moderate mold conditions. Conversely, under other conditions, the concentrations of these substances are relatively low.

#### 3.1.6. Natural Mildew

Figure 1F presents the analysis of sensor response values for naturally moldy soybean samples at various mold levels. The sensors with the highest response values are W1W (sensitive to inorganic sulfides) and W2W (sensitive to organic sulfides), followed by W5S (highly sensitive to nitrogen oxides), W1S (sensitive to short-chain alkanes such as methane), and W2S (sensitive to alcohols, ethers, aldehydes, and ketones). However, under severe mold conditions, the responses of all sensors are weak, with deviation values below 4. This indicates that while the concentrations of organic sulfides, inorganic sulfides, and nitrogen compounds are relatively high in naturally moldy soybean samples, they are comparatively low under severe mold conditions.

The analysis indicates that the ten sensors of the electronic nose (E-nose) exhibit significant responses to the volatile substances in moldy soybean samples. Response values primarily vary between 0 and 20 s, level off from 20 to 70 s, and stabilize thereafter. The response values of sensors for soybean samples inoculated with *Aspergillus niger* are generally higher than those of other samples. The response trends for samples inoculated with *Penicillium rubens* and naturally moldy soybeans are similar, showing stronger responses in mild mold conditions. Similarly, the response trends for samples inoculated with *Rhizopus microsporus* and *Aspergillus versicolor* display strong responses in both mild and severe mold states. For soybean samples inoculated with *Penicillium oxalicum*, the sensor responses are weak in all states except for mild mold. Additionally, all moldy soybean samples exhibit weak sensor responses in the severe mold state. During their growth and reproduction, the fungi in the soybean samples significantly consume nutritional components, such as proteins and fats, while generating metabolites like alcohols, ketones, and organic sulfides. This leads to distinct sensor array responses at different mold levels. In the later stages of mold growth, as the nutritional components of the soybean are nearly depleted, fungal growth and metabolism are inhibited, resulting in reduced metabolite production [41]. Consequently, in the severe mold state, sensor response values for samples inoculated with the five predominant fungi and naturally moldy soybeans are lower, indicating that the concentrations of various substances are relatively low at this time.

An analysis of all response value graphs concludes that the sensors with the strongest responses throughout the testing process are primarily W2W (sensitive to organic sulfides) and W1W (sensitive to inorganic sulfides), followed by W5S (sensitive to nitrogen oxides), W1S (sensitive to methane and short-chain alkanes), and W2S (sensitive to alcohols, ethers, aldehydes, and ketones), while the responses of other sensors are relatively weak. This indicates that the main volatile substances that change during soybean mold include organic sulfides, inorganic sulfides, nitrogen oxides, short-chain alkanes, as well as alcohols, aldehydes, and ketones.

### 3.2. Radar Map Analysis of Soybeans with Different Degrees of Mildew

**Figure 2:**
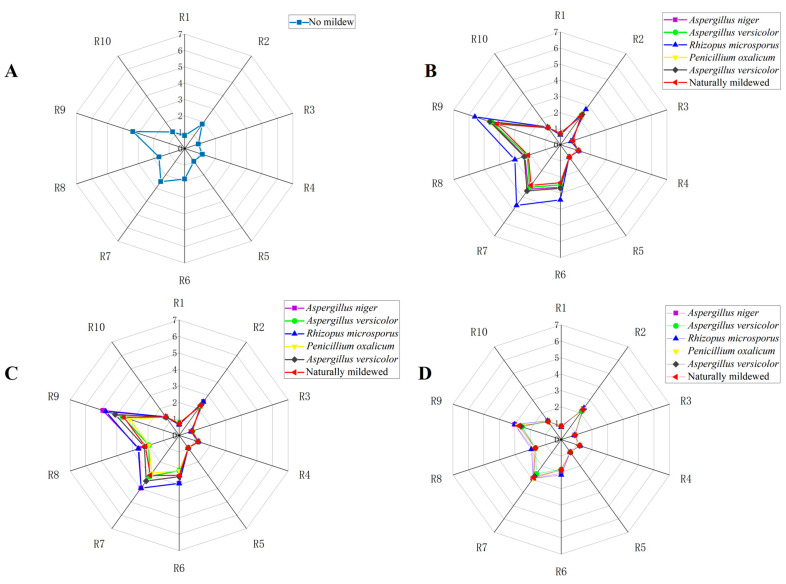
Comparative analysis of response values of soybean sensors with different molds. (**A**) No mildew; (**B**) Mild mildew; (**C**) Moderate mildew; (**D**) Severe mildew.

Since the sensor signal responses of the electronic nose (E-nose) stabilize around 70 s, the average response values at 70 s, 71 s, and 72 s are extracted as feature data for subsequent statistical analysis. Figure 2 presents a radar analysis of the average response values from the E-nose for soybean samples with varying degrees of mold. This analysis illustrates significant differences in overall sensor responses across different mold levels, particularly in R9 (W2W, sensitive to organic sulfides) and R7 (W1W, sensitive to inorganic sulfides). These differences indicate substantial concentration variations of organic and inorganic sulfides in soybean samples at various mold stages, which can be regarded as key characteristic substances for identifying soybean mold. Furthermore, it was observed that, at the same mold level, the sensor responses for the five predominant fungal inoculations and naturally moldy soybean samples exhibit certain similarities. However, the sensor responses for soybean samples inoculated with *Rhizopus microsporus* differ significantly from those of other samples. This variation can be analyzed in conjunction with the sensory characteristics of soybeans during the molding process, primarily due to the higher total fungal count in the samples inoculated with *Rhizopus microsporus*. This increased fungal count results in a larger colony coverage area, significantly affecting the sensor responses.

### 3.3. Identification and Analysis of Mold Inoculation and Naturally Moldy Soybean

PCA is a widely used method for processing E-nose data, effectively reducing data dimensionality by transforming correlated variables into principal component factors. This transformation enables the assessment of sample differences based on the contribution rates of these factors. LDA is another effective dimensionality reduction technique in pattern recognition, which maximizes distinguishability by preserving the direction that minimizes the ratio of within-class to between-class variance. In this experiment, feature values from the ten sensors are extracted and analyzed for their contribution rates, followed by PCA and LDA analyses on the feature values from the five predominant fungal inoculations and naturally moldy soybean samples.

#### 3.3.1. E-Nose Sensor Contribution Rate Analysis

**Figure 3:**
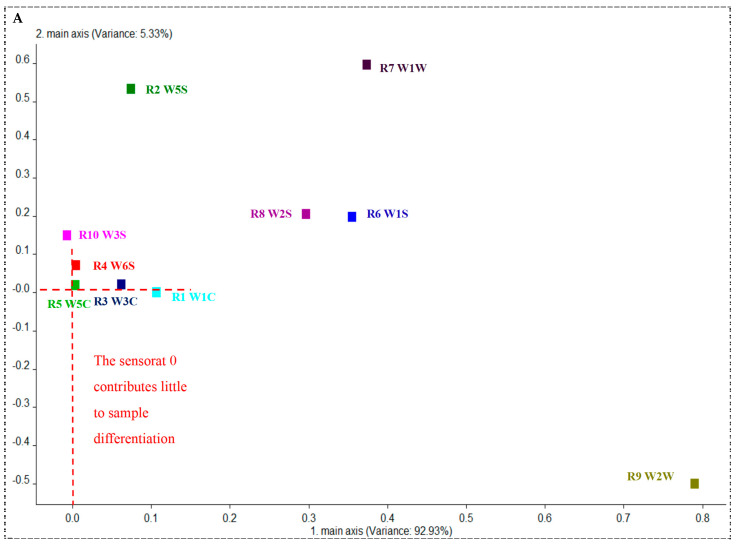

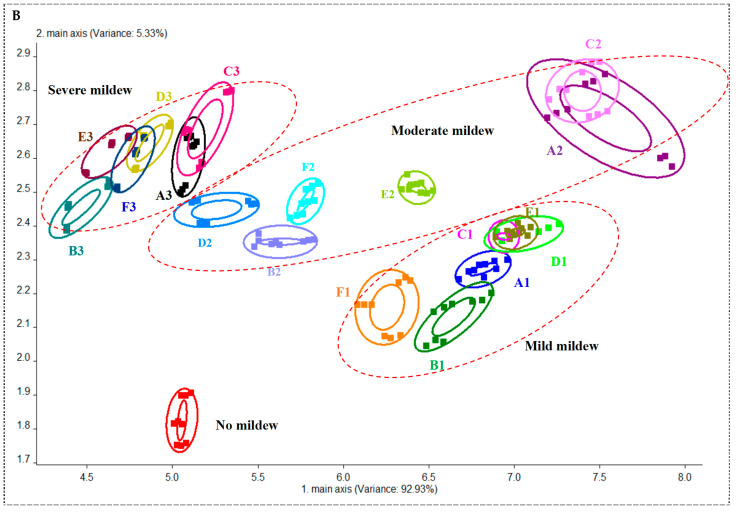

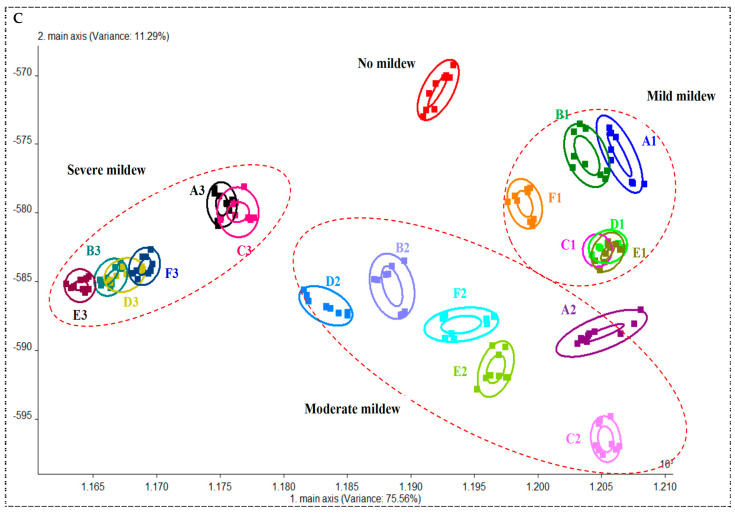
Sensor contribution: PCA and LDA score plots. (**A**) Analysis chart of the sensor contribution rate. (**B**) PCA scores of five dominant molds inoculated and naturally mildewed soybeans. (**C**) LDA scores of five dominant molds inoculated and naturally mildewed soybeans. Note: A–E are *Aspergillus niger*, *Penicillium rubrum*, *Rhizopus microsporioides*, *Penicillium oxalicum*, and *Aspergillus versicolor* inoculated soybeans, and F is naturally mildewed soybeans; 1–3 represent mild, moderate, and severe mildew respectively.

Figure 3A illustrates the contribution rate analysis of the ten E-nose sensors using the Loadings algorithm. The contribution rate of the first principal component is 92.93%, while that of the second principal component is 5.33%, indicating that the primary differences among the sensors are reflected in the first principal component. Notably, the W2W sensor (sensitive to organic sulfur compounds) contributes the most to the first principal component, followed by W1W (sensitive to inorganic sulfur compounds), W1S (sensitive to methane and other short-chain alkanes), and W2S (sensitive to alcohols, ethers, aldehydes, and ketones). The sensors significantly contributing to the second principal component are W1W and W5S (sensitive to small molecular nitrogen compounds). These results suggest that during the molding process, soybeans produce substantial amounts of both inorganic and organic sulfur compounds, along with smaller quantities of alcohols, ethers, aldehydes, ketones, nitrogen compounds, and short-chain alkanes such as methane.

#### 3.3.2. PCA of Mold Inoculation and Natural Mildew Soybean

To analyze the differences among the five fungal-inoculated and naturally moldy soybean samples, principal component analysis (PCA) was conducted on their E-nose feature values. As shown in Figure 3B, the combined contribution rates of the first and second principal components are 98.26%, indicating that they capture the vast majority of the information from the feature data. The contribution rate of the first principal component is 92.93%, while that of the second is 5.33%, suggesting that the differences among the moldy soybean samples are primarily reflected in the first component. Notably, there is significant overlap among the points representing the five fungal-inoculated and naturally moldy soybean samples, indicating that PCA does not effectively distinguish the feature values and cannot accurately identify the different samples.

#### 3.3.3. LDA of Mold Inoculation and Natural Mildew Soybean

To effectively distinguish the five fungal-inoculated and naturally moldy soybean samples, linear discriminant analysis (LDA) was performed on their E-nose feature values (Figure 3C). The results show that LDA successfully reduces within-group differences, making the distinctions among the five sample points clear. The distribution areas of samples from the same fungal inoculation (or naturally moldy) are concentrated, with only minimal overlap. The overall classification accuracy achieved is 86.85%, indicating that LDA can effectively differentiate between the five fungal-inoculated and naturally moldy soybean samples.

### 3.4. Identification and Analysis of Soybean with Different Mildew Grades

#### 3.4.1. PCA of Soybean with Different Mildew Grades

**Figure 4:**
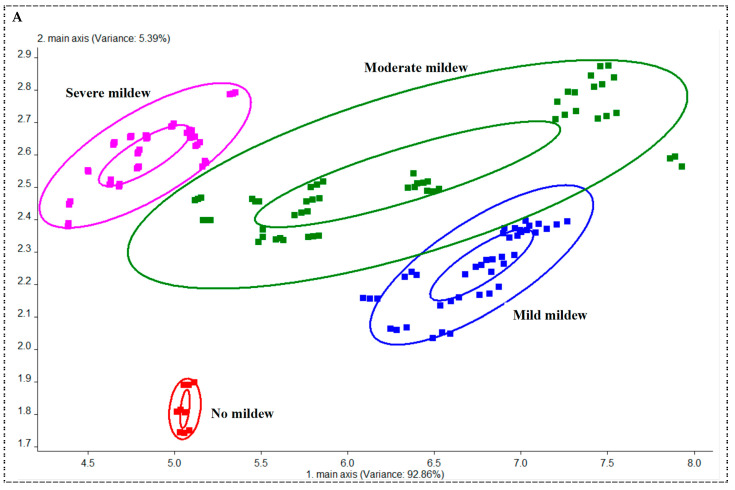

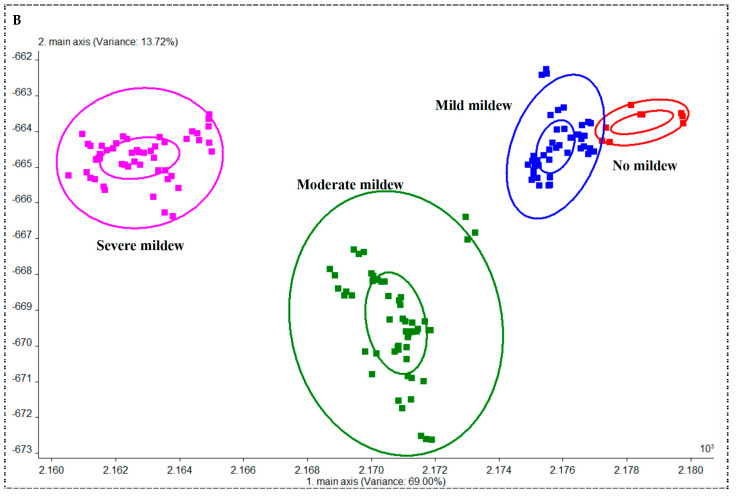
PCA and LDA scores of different moldy soybean grades. (**A**) PCA score map; (**B**) LDA score map.

Figure 4A displays the PCA score plot for soybean samples at various mold levels, showing that the first and second principal components account for 98.25% of the data variance, with the first component alone contributing 92.86%. The clear separation between non-moldy and moldy soybean samples indicates that PCA is effective in distinguishing mold presence. Additionally, the sample points for light, moderate, and severe mold are grouped distinctly along the X-axis, with no overlap, demonstrating a clear pattern. These findings suggest that PCA can successfully differentiate soybean samples based on mold levels.

#### 3.4.2. LDA of Soybean with Different Mildew Grades

LDA analysis, as shown in Figure 4B, demonstrates superior discrimination compared to PCA for soybean samples at different mold levels. Sample points corresponding to the same mold level are more tightly clustered, while those from different levels show significant separation. The sequential arrangement of light, moderate, and severe mold samples along the X-axis indicates a clear pattern. With an overall classification accuracy of 82.72%, these results confirm that LDA is effective in distinguishing between soybeans based on their mold levels.

## 4. Discussion

This study simulated soybean spoilage during transportation by isolating five fungal species and inoculating them onto soybeans. The spoiled soybeans were classified into four levels: no spoilage, mild spoilage, moderate spoilage, and severe spoilage. Fungi produce various volatile compounds, such as alcohols, ketones, and aldehydes, during growth, metabolism, and reproduction, which contribute to off-odors. The degree of fungal contamination is correlated with the intensity of these off-odors [42]. Therefore, E-nose technology can be used to distinguish and classify the spoilage levels of soybeans.

Currently, extensive research has been conducted on using E-nose technology to assess fungal contamination in grains. Huang et al. [43] developed a static E-nose-based model for classifying maize spoilage, using Principal Component Analysis (PCA) and Linear Discriminant Analysis (LDA) to differentiate between healthy and spoiled kernels. The results showed that PCA had poor differentiation performance for healthy and spoiled maize, while LDA provided better discrimination, achieving an accuracy rate of 81.1%. Shen et al. [44] applied E-nose technology to detect peanuts contaminated by five harmful fungi and analyzed their spoilage state during storage. Sensor response signals and weight analysis indicated that after fungal contamination, peanuts produced higher amounts of aromatic, hydrocarbon, amine, and alkane compounds. PCA and LDA analyses showed that E-nose effectively differentiated between various spoilage states of peanuts during storage. The longer the storage period, the better the differentiation, with an accuracy rate of 87.5% on day 9 of spoilage. The findings are consistent with previous research; however, this study did not integrate other detection methods and therefore did not conduct qualitative and quantitative analyses of specific components in spoiled soybeans. The changes in the relative content of compounds, particularly their relationship with the degree of spoilage and the types of fungi, provide a valuable complement to E-nose technology.

## 5. Conclusions

This study investigates the applicability of electronic nose (E-nose) technology for detecting spoilage in soybeans. The results reveal that the sensor with the highest contribution in the E-nose is W2W, which is sensitive to organic sulfur compounds, followed by W1W (sensitive to inorganic sulfur compounds), W5S (sensitive to small molecular nitrogen compounds), W1S (sensitive to methane and other short-chain alkanes), and W2S (sensitive to alcohols, ethers, aldehydes, and ketones). These findings suggest that the differences in volatile substances among the spoiled soybean samples are predominantly associated with organic sulfur compounds, with notable variations also observed in inorganic sulfur, nitrogen compounds, and short-chain alkanes such as methane, alcohols, ethers, aldehydes, and ketones. The results of the principal component analysis (PCA) and linear discriminant analysis (LDA) demonstrate that both methods exhibit moderate discrimination effectiveness for differentiating between various fungal inoculations and naturally spoiled soybean samples, though they perform better in distinguishing between soybean samples of varying spoilage levels, achieving an LDA classification accuracy of 82.72%. In conclusion, E-nose technology can effectively identify spoilage levels in soybeans, providing valuable theoretical data for the development of fungal detection methods and enhancing quality and safety measures.

## Figures and Tables

**Table 1:** Performance of the sensor arrays of the PEN3 electronic nose.

No. in Array	Sensor Name	Reaction Compound	Typical Target
R1	W1C	Aromatic compounds	C_6_H_5_CH_3_
R2	W5S	Oxynitride	NO_2_
R3	W3C	Aromatic constituents, mainly ammonia	C_6_H_6_
R4	W6S	Hydrogen	H_2_
R5	W5C	Alkanes, aromatic compounds	C_3_H_8_
R6	W1S	Broad methane	CH_4_
R7	W1W	Sulfides and organic sulfides	H_2_S
R8	W2S	Broad alcohols	C_2_H_5_OH
R9	W2W	Aromatics, organic sulfides	H_2_S
R10	W3S	Alkanes, especially methane	CH_4_

## Data Availability

The original contributions presented in this study are included in the article. Further inquiries can be directed to the corresponding authors.
